# Salt coatings functionalize inert membranes into high-performing filters against infectious respiratory diseases

**DOI:** 10.1038/s41598-020-70623-9

**Published:** 2020-08-17

**Authors:** Ilaria Rubino, Euna Oh, Sumin Han, Sana Kaleem, Alex Hornig, Su-Hwa Lee, Hae-Ji Kang, Dong-Hun Lee, Ki-Back Chu, Surjith Kumaran, Sarah Armstrong, Romani Lalani, Shivanjali Choudhry, Chun Il Kim, Fu-Shi Quan, Byeonghwa Jeon, Hyo-Jick Choi

**Affiliations:** 1 Department of Chemical and Materials Engineering, University of Alberta, Edmonton, AB T6G 1H9 Canada; 2 School of Public Health, University of Alberta, Edmonton, AB T6G 1H9 Canada; 3 Department of Biomedical Science, Graduate School, Kyung Hee University, Seoul, 130-701 South Korea; 4 Department of Mechanical Engineering, University of Alberta, Edmonton, AB T6G 1H9 Canada; 5 Department of Medical Zoology, Kyung Hee University School of Medicine, Seoul, 130-701 South Korea; 6 Environmental Health Sciences, School of Public Health, University of Minnesota, Saint Paul, MN 55108 USA

**Keywords:** Disease prevention, Public health, Infectious diseases, Bacterial infection, Biomaterials, Respiratory tract diseases, Nanobiotechnology, Design, synthesis and processing

## Abstract

Respiratory protection is key in infection prevention of airborne diseases, as highlighted by the COVID-19 pandemic for instance. Conventional technologies have several drawbacks (i.e., cross-infection risk, filtration efficiency improvements limited by difficulty in breathing, and no safe reusability), which have yet to be addressed in a single device. Here, we report the development of a filter overcoming the major technical challenges of respiratory protective devices. Large-pore membranes, offering high breathability but low bacteria capture, were functionalized to have a uniform salt layer on the fibers. The salt-functionalized membranes achieved high filtration efficiency as opposed to the bare membrane, with differences of up to 48%, while maintaining high breathability (> 60% increase compared to commercial surgical masks even for the thickest salt filters tested). The salt-functionalized filters quickly killed Gram-positive and Gram-negative bacteria aerosols in vitro, with CFU reductions observed as early as within 5 min, and in vivo by causing structural damage due to salt recrystallization. The salt coatings retained the pathogen inactivation capability at harsh environmental conditions (37 °C and a relative humidity of 70%, 80% and 90%). Combination of these properties in one filter will lead to the production of an effective device, comprehensibly mitigating infection transmission globally.

## Introduction

Airborne pathogens, including bacteria and viruses, transmit in the environment in the form of droplets (> 5 µm) or aerosols (< 5 µm)^1^. Due to long travelling distance and respirability of aerosols, airborne transmission can occur very easily^2^. As such, respiratory protection measures are essential first lines of defense in health care settings, congregate settings (e.g., correctional facilities, military barracks, homeless shelters, refugee camps, dormitories, and nursing homes), households (including family members and caregivers), and in the event of pandemic or epidemic outbreaks. As the World Health Organization (WHO) and scientific community highlight the urgency in stopping infectious diseases and preparing for the next disease outbreaks^3^, and new pandemic strains such as COVID-19 emerge, development of effective, readily available infection control measures is recognized as a primary challenge in health care.

Specifically, in health care facilities, bacteria including *Klebsiella pneumoniae *(*K. pneumoniae*), *Staphylococcus aureus* (*S. aureus*), *Pseudomonas aeruginosa *(*P. aeruginosa*), *Streptococcus pyogenes* (*S. pyogenes*) and *Escherichia coli *(*E. coli*) are the major causes of nosocomial (hospital-associated) infections^4,5^. Bacteria can transmit infections through the air in locations such as operating theatres, corridors, waste containers as well as intensive care, burn and orthopedic units^5–9^. Nosocomial *K. pneumoniae* infections have mortality rates as high as 50%; additionally, the WHO has reported global resistance to third-generation cephalosporins and carbapenem in 30–60% and up to 50% of cases, respectively^10^. Methicillin-resistant *S. aureus* (MRSA), also transmissible through the air^11^, constitutes another major pathogen in nosocomial infections, causing 20% to above 80% of *S. aureus* nosocomial infections worldwide^10^.

The most commonly used respiratory protective device in health care is the N95 respirator, which is designed to capture aerosols. Surgical masks, commonly used to block large droplets during surgeries, were historically utilized against bioaerosols. Surgical masks have seen a continued use for this purpose in the general public during more recent outbreaks, in spite of the improper application^12–15^. This is due to their high availability, affordability and comfort. Although respirators and masks play a critical role in the protection against bioaerosols, they are limited by four major technical issues of the filters: (i) cross-infection, (ii) filtration efficiency, (iii) breathability, and (iv) recyclability^16^. When respirators/masks capture bioaerosols, they become contaminated, since pathogens survive on the surface of the filters. This presents a threat to the wearer and people they come in contact with. As the devices can easily become a source of infection, commercial respirators/masks are limited to a single use. Additionally, in the traditional technologies, it is well-known that increasing the filtration efficiency leads to filters that cause higher pressure drop across the masks, with consequent difficulty in breathing. Some efforts have been directed towards production of filters that can overcome the burden of decreased breathability^17,18^. However, these methods cannot address the risks arising from the survival of pathogens captured on the filters.

We envisioned the development of a technology that could solve all major technical challenges simultaneously, which cannot be addressed by conventional respiratory protective devices. The mechanism we developed consists of functionalizing the polypropylene (PP) fibers of large-pore (~ 60 μm) membranes with salt. Large-pore membranes have high breathability compared to commercial respirators/masks, but no pathogen filtration activity. We hypothesized that the salt coating would: (i) increase the filtration efficiency of the breathable membranes, turning them into an active filtration unit, and (ii) inactivate bacteria. Our previous report showed that coating the filters of surgical masks with sodium chloride (NaCl) salt kills multiple strains of influenza virus; additionally, a significant increase in the filtration efficiency of viral aerosols on the NaCl-coated filters compared to the bare surgical mask filters was observed^19^. In this work, non-functional large-pore membranes were coated with three types of salt [NaCl, potassium sulfate (K_2_SO_4_) and potassium chloride (KCl)]. We demonstrate that salt functionalization greatly increases the filtration efficiency of large-pore membranes, while at the same time exhibiting no significant increase in pressure drop. The proposed filtration mechanism is non-mechanical in nature, which is evidenced by the insensitivity of filtration efficiency with respect to increasing layer numbers and the high filtration efficiency of the top layer (the first layer interacting with aerosols) of salt-functionalized filters even with presence of large-pore fiber mesh. Additionally, the salt recrystallization upon exposure of the salt-functionalized filters to infectious aerosols caused the physical damage of the bacteria independent of strain (*K. pneumoniae,* MRSA*, P. aeruginosa, S. pyogenes* and *E. coli*), leading to their inactivation. As such, we report a diverse respiratory protection system that is manufactured without extensive engineering efforts, and achieves quick universal pathogen inactivation, high filtration efficiency, high breathability, and safe recyclability, all in one platform.

## Results and discussion

### Production and characterization of salt-coated filters

Development of environment-resistant filters is key to their universal application. Thus, when designing the salt-functionalized filters, fine-tuning of the properties of the pathogen-inactivating mask to satisfy different temperature/humidity conditions during use and storage was considered. To this end, safe and inexpensive salt types with different critical relative humidity (RH) were selected. At a given temperature, if a salt is exposed to an RH above its critical RH, it will take up water from the atmosphere. NaCl, KCl and K_2_SO_4_ have critical RH of 74.7%, 81.2% and 95.7% at 40 °C, respectively^20^, and were investigated in this study. The salt coatings were applied onto the PP microfibers of large-pore membranes, which were not designed to provide protection against bacteria aerosols. The membranes (bare membranes) were dried in different volumes of coating solution (V_salt_; NaCl, K_2_SO_4_ or KCl), to produce filters coated with different amounts of salts per unit area (W_salt_). The linear relationship between W_salt_ and V_salt_ for each salt type at different thicknesses can be found in Supplementary Fig. S1a–c (Pearson test, *P* values on graphs).

**Figure 1:**
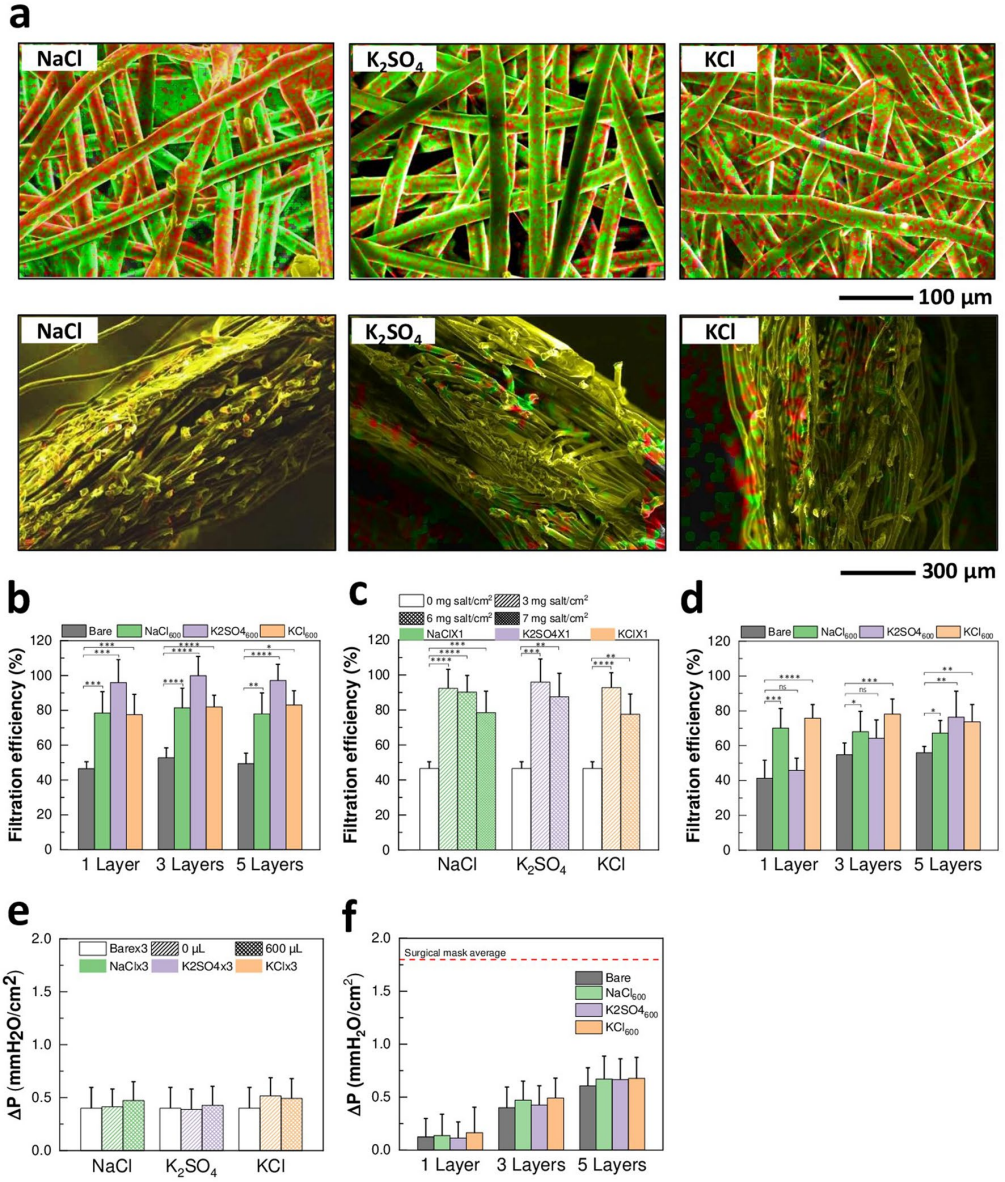
Characterization and performance of salt-coated filters. (**a**) EDX mapping images of NaCl (left—combination of Na (red) and Cl (green) mapping images), K_2_SO_4_ [center—combination of K (red) and S (green)], and KCl [right—combination of K (red) and Cl (green)] filters, showing formation of NaCl, K_2_SO_4_, and KCl coatings, respectively. Top: plain view, bottom: cross-sectional view. (**b**) Filtration efficiency of Bare, NaCl_600_, K_2_SO_4_ _600_, and KCl_600_ with 1, 3 and 5 stacked layers with no air flow [*n* = 7–20, mean ± standard deviation (SD)]. (**c**) Filtration efficiency of Bare × 1, and NaCl × 1, K_2_SO_4_ × 1, and KCl × 1 coated with 3, 6 and 7 mg salt/cm^2^ with no air flow (*n* = 7–15, mean ± SD). (**d**) Filtration efficiency of Bare, NaCl_600_, K_2_SO_4_ _600_, and KCl_600_ with 1, 3 and 5 stacked layers at an air flow rate of 15 Lpm (*n* = 10, mean ± SD). (**e**) Pressure drop of Bare × 3, and NaCl × 3, K_2_SO_4_ × 3, and KCl × 3 coated with different amount of salt (*n* = 26–45, mean ± SD). No star: ns. (**f**) Pressure drop of Bare, NaCl_600_, K_2_SO_4_ _600_, and KCl_600_ with 1, 3 and 5 stacked layers (*n* = 27–65, mean ± SD). No star: ns. Dotted line: average pressure drop of commercial surgical mask. For all panels: ns: *P* > 0.05; *P < 0.05; **P < 0.01; ***P < 0.001; ****P < 0.0001, by ANOVA.

By stacking different numbers of membrane layers during drying, the overall thickness was varied. This allows for control over the final design of the salt-functionalized filters based on application needs. The formation of NaCl, K_2_SO_4_ or KCl salt coatings was analyzed by scanning electron microscopy (SEM) and energy dispersive X-ray (EDX) mapping. Homogenous coating formation on the surface of the fibers and throughout the cross section of multi-layer filters was observed (Fig. 1a and Supplementary Fig. S1d). These results show the successful fabrication of filters uniformly functionalized with NaCl, K_2_SO_4_ or KCl salts at controlled thicknesses.

### Filtration efficiency against bacteria aerosols

The bacteria filtration performance of the salt-coated filters was probed against *K. pneumoniae* aerosols at 0 and 15 Lpm air flow rates. Overall, at 0 Lpm air flow, salt-coated filters showed significantly higher filtration efficiency than bare membranes at all conditions of salt type (NaCl, K_2_SO_4_ and KCl), thickness (1, 3 or 5 layers), and salt amount (3, 6 or 7 mg/cm^2^) (two-way ANOVA, *P* values on graphs) (Fig. 1b,c). In particular, the NaCl, K_2_SO_4_ and KCl filters exhibited an increase in filtration efficiency from 50 to 79%, 98% and 81%, respectively, as compared to bare membranes (Fig. 1b). Even when the *K. pneumoniae* aerosols were passed through the filters at 15 Lpm, NaCl and KCl salt filters still captured significantly more bacteria than bare membranes; 5-layered K_2_SO_4_ filters showed a significant increase in filtration efficiency compared to bare membranes as well (two-way ANOVA, *P* values on graphs) (Fig. 1d). Interestingly, with the sole exception of K_2_SO_4_ filters tested at 15 Lpm air flow, the filtration efficiency of the salt-coated filters did not depend on the filter thickness (two-way ANOVA, *P* > 0.05) (Fig. 1b,d). This led us to hypothesize that the top layer (first layer to interact with the aerosols) is mainly responsible for the increased filtration efficiency observed in salt-coated filters. Considering the relatively large size of pores against aerosols, the collection of aerosols possibly occurs via the absorption mechanisms of coated dry-salt agents (mainly those applied on the top layer), as opposed to the conventional filtration mechanism where the collection efficiency is governed by the fine mesh size and the number of added layers.

### Pressure drop across the salt-coated filters

As traditional technologies that enhance capture of pathogens/particles negatively affect the breathability of the filters, the pressure drop of the salt-coated filters at an air flow rate of 8 Lpm was measured next. Pressure drop levels evaluate the perceived resistance breathing through the filters. Notably, the pressure drop across NaCl, K_2_SO_4_ and KCl filters did not significantly change as compared to bare membranes, irrespective of the amount of coated salt (one-way ANOVA, *P* > 0.05) (Fig. 1e) and the thickness of the filters (two-way ANOVA, *P* > 0.05) (Fig. 1f). Since the pore size of the fiber mesh remains sufficiently large after the salt coating treatment (Supplementary Fig. S1e and Supplementary Table S2), the above results further suggest that the proposed filtration mechanism is non-mechanical in nature, ensuring maximum filtration efficiency with high breathability.

To represent the overall filter performance, the quality factors (QF) of the salt-coated filters were compared with those of the bare membranes at 15 Lpm. The QF values are reported in Supplementary Table S1 and represent the ratio between the filtration efficiency and the pressure drop of filters^1^. The QF of NaCl, K_2_SO_4_ and KCl filters increased by 107%, 26% and 103%, respectively, compared with bare membranes at 1-layer thickness, 16%, 23% and 57% for 3 layers and 23%, 66% and 51% for 5 layers. The increase in QF of the salt-functionalized filters compared to bare membranes indicates an overall enhancement in performance. It is also important to note that, in theory, the QF is independent of the filter thickness. However, NaCl and KCl filters show differences in QF at different thicknesses. This is related to the fact that the top layer constitutes the main capture medium responsible for increased filtration efficiency of the salt-coated filters compared to bare membranes, as mentioned. Overall, these performance test results indicate that functionalizing large-pore membranes with salts leads to a substantial increase of the amount of captured bacteria, without increasing the resistance to air flow, yielding improved filter quality.

### Inactivation of *K. pneumoniae* on the salt-coated filters and protective efficacy in vivo

**Figure 2:**
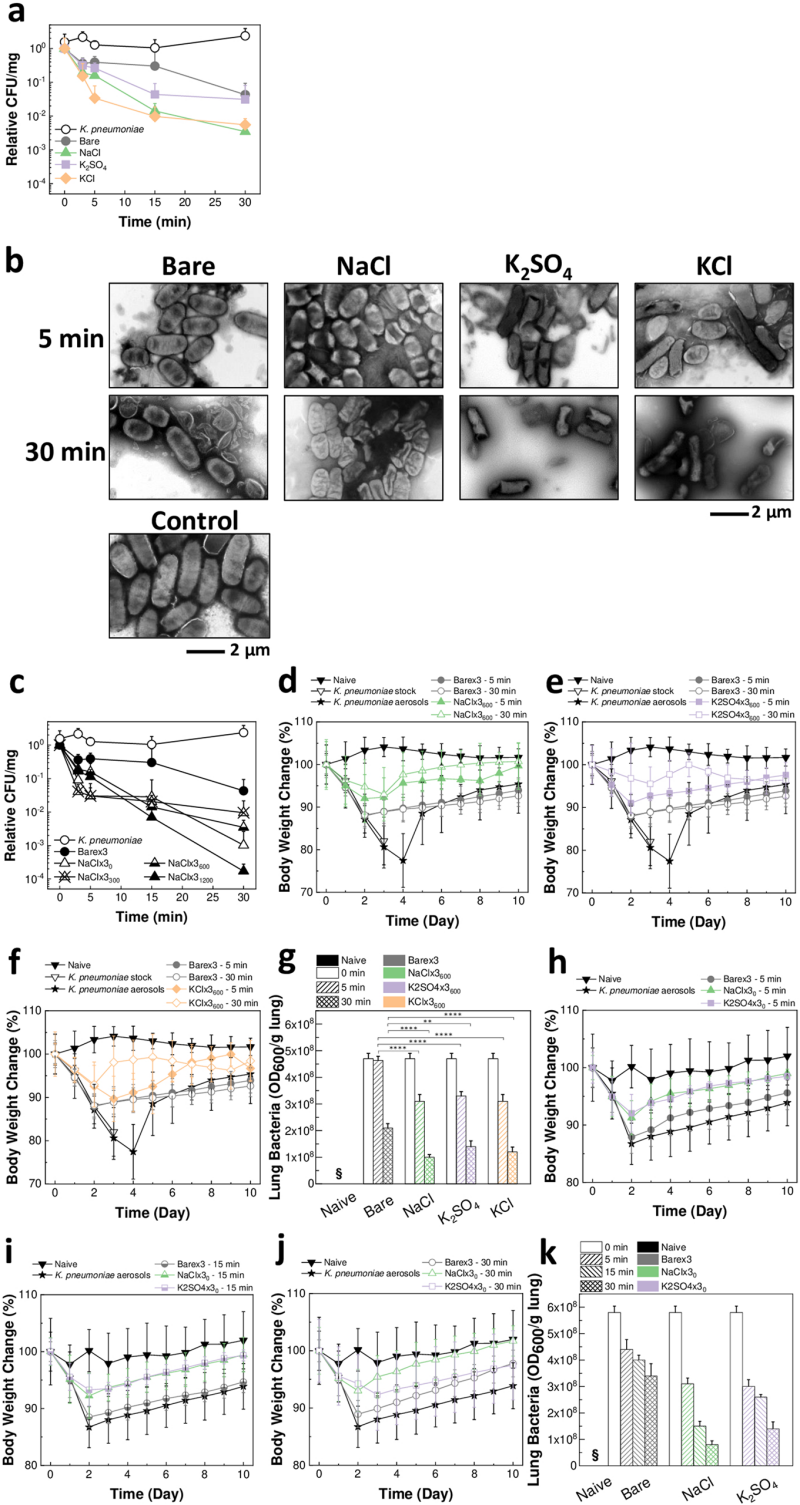
Pathogen inactivation on salt-coated filters due to salt recrystallization. (**a**) CFU change showing the effect of incubation time on *K. pneumoniae* exposed to bare membranes and NaCl, K_2_SO_4_, and KCl filters (*n* = 5–38, mean ± SD). (**b**) TEM images of *K. pneumoniae* incubated on Bare × 3, NaCl × 3_600_, K_2_SO_4_ × 3_600_, and KCl × 3_600_ for 5 and 30 min (top), and of *K. pneumoniae* suspension as control (bottom). (**c**) CFU change showing the effect of incubation time on *K. pneumoniae* exposed to NaCl filters coated with different amount of salt (*n* = 5–38, mean ± SD). (**d**–**g**) Mice body weight change after infection with bacteria incubated on bare membranes, and NaCl × 3_600_ (**d**), K_2_SO_4_ × 3_600_ (**e**), and KCl × 3_600_ (**f**) for 5 and 30 min (*n* = 3–8, mean ± SD), and OD_600_ of lungs (*n* = 3–8, mean ± SD; §: below detection limit) (**g**). **P < 0.01; ****P < 0.0001, by one-way ANOVA. (**h–k**) Mice body weight change after infection with bacteria incubated on bare membranes, NaCl × 3_0_, and K_2_SO_4_ × 3_0_ for 5 min (**h**), 15 min (**i**), and 30 min (**j**) (*n* = 4–8, mean ± SD), and OD_600_ of lungs (*n* = 3–4, mean ± SD; §: below detection limit) (**k**).

The pathogen inactivation on NaCl, K_2_SO_4_ and KCl filters was investigated by exposing them to *K. pneumoniae* bacterial aerosols. Time-dependent inactivation of bacteria incubated on filters coated with all salt types was observed (General Linear Model, *P* < 0.001) (Fig. 2a). In particular, the KCl-coated filters exhibited significant colony forming units (CFU) reduction compared to bare membranes even within 5 min from aerosol exposure (two-way ANOVA, *P* ≤ 0.0001). As shown in Fig. 2b and Supplementary Fig. S2a, in contrast to intact bacteria in the control and on bare membranes, bacteria recovered from the salt-coated filters were found to be severely damaged due to the salt growth during the evaporation process. This explains the destabilization of the bacteria measured by CFU. Furthermore, the effect of different amounts of NaCl coated on the filters on the stability of *K. pneumoniae* aerosols was investigated. NaCl-coated filters showed rapid time-dependent bacteria inactivation (General Linear Model, *P* < 0.001) (Fig. 2c). Notably, NaCl × 3_1200_ caused a 4 log CFU reduction within 30 min from aerosol exposure. The TEM analysis revealed the rapture, structural damage and morphological changes incurred by the bacteria due to the salt recrystallization (Supplementary Fig. S2b). Similarly, when *K. pneumoniae* aerosols were exposed to the K_2_SO_4_ and KCl filters prepared to contain a lower amount of salt (K_2_SO_4_ × 3_0_ and KCl × 3_0_), quick time-dependent CFU reduction was still measured (General Linear Model, *P* < 0.001) (Supplementary Fig. S3a,b)_._ The bacteria inactivation on K_2_SO_4_ × 3_0_ and KCl × 3_0_ was also confirmed in the TEM analysis (Supplementary Fig. S3c). In general, it is worth noting that the actual infectious aerosols will have significantly lower levels of bacterial concentration than the exposure aerosols used in the tests; as such, it is expected that further pathogen inactivation will be observed in real-life scenarios. Overall, the bacteria stability results indicate that the NaCl, K_2_SO_4_ and KCl coatings rapidly neutralize bacteria by physical damage induced during the salt recrystallization process.

The bacteria inactivation findings were confirmed in vivo. Mice were infected with *K. pneumoniae* recovered from NaCl × 3_600_, K_2_SO_4_ × 3_600_ and KCl × 3_600_ at 5 and 30 min incubation. At 30-min incubation, the bare membrane group reached a body weight loss that was significantly higher than that of NaCl, K_2_SO_4_ and KCl filter groups by 10% (Fig. 2d–f) (two-way ANOVA, *P* ≤ 0.05). As shown in Fig. 2g, OD_600_ measured from the lungs of salt filter group mice were significantly lower than those of the control groups (bare membranes) (one-way ANOVA, *P* values on graph). Furthermore, the OD_600_ levels decreased with incubation time (General Linear Model, *P* < 0.001). After 2–4 days, all salt filter mice groups showed a significant rapid recovery in body weight as opposed to the bacteria aerosol control group (two-way ANOVA, *P* ≤ 0.05), unlike bare membrane groups (Fig. 2d–f). Additionally, the in vivo response to *K. pneumoniae* was investigated after incubation on the NaCl × 3_0_ and K_2_SO_4_ × 3_0_ for 5, 15 and 30 min. At day 2 post-infection, the salt filter groups exhibited a significantly lower body weight loss than the bacteria aerosol control group (two-way ANOVA, *P* ≤ 0.05), unlike the bare membrane group (Fig. 2h–j). The lung OD_600_ measurements further supported these results by showing significantly lower levels in NaCl × 3_0_ and K_2_SO_4_ × 3_0_ mice groups than bare membrane groups (one-way ANOVA, *P* ≤ 0.0001) (Fig. 2k). In general, a decrease in lung OD_600_ was also observed with the increase in incubation time on the filters (General Linear Model, *P* < 0.001). Thus, these studies support the in vitro results, indicating that protection of the mice was obtained by the NaCl, K_2_SO_4_ and KCl salt coatings due to inactivation of the aerosolized bacteria.

### Strain-independent inactivation of bacteria on salt-coated filters

**Figure 3:**
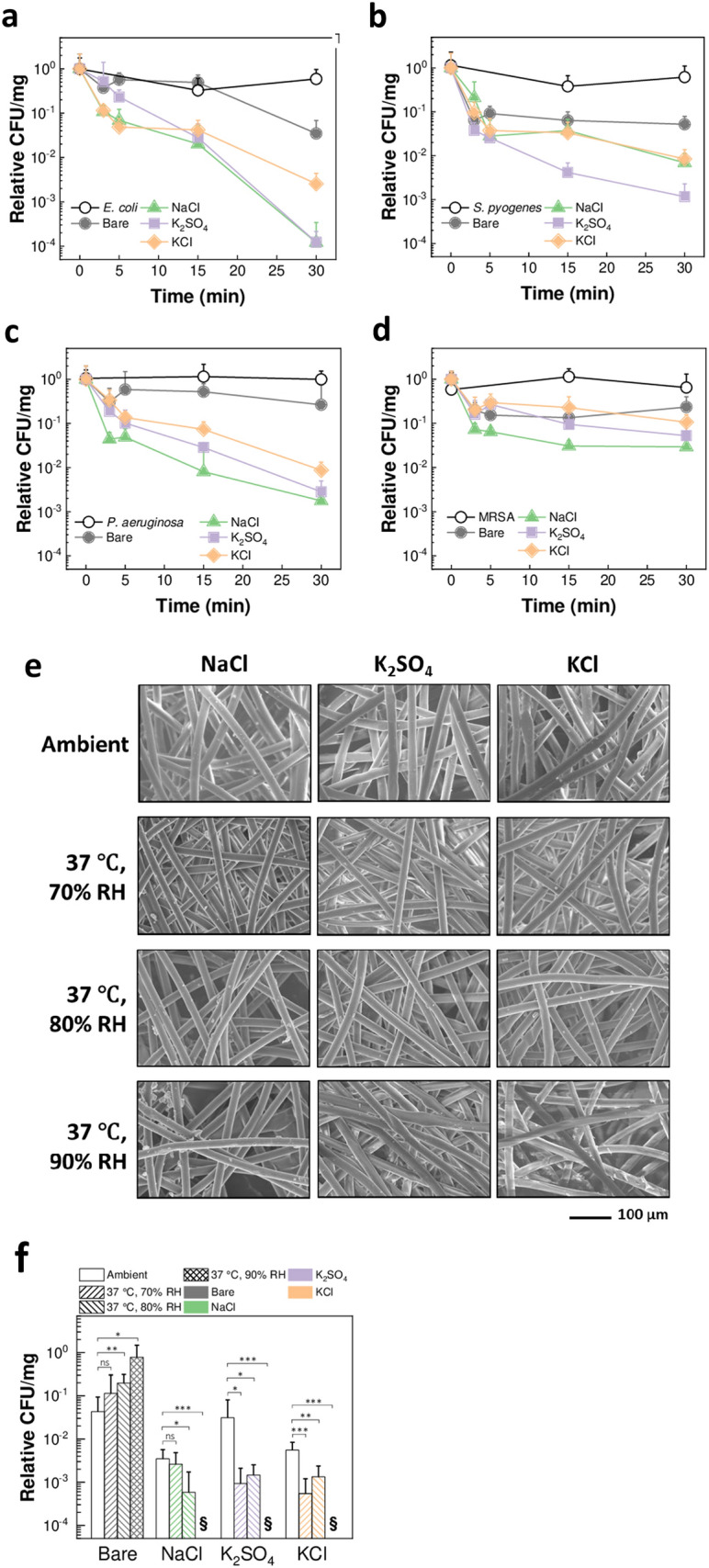
Strain-nonspecific protective efficacy and environmental stability. (**a**–**d**) CFU change showing the effect of incubation time on *E. coli* (**a**), *S. pyogenes* (**b**), *P. aeruginosa* (**c**), and MRSA (**d**) exposed to bare membranes and NaCl, K_2_SO_4_, and KCl filters (*n* = 4–19 for (**a**), *n* = 3–19 for (**b**), *n* = 3–25 for (**c**), *n* = 2–10 for (**d**), mean ± SD). (**e**) SEM images of NaCl × 3_600_, K_2_SO_4_ × 3_600_, and KCl × 3_600_ at ambient condition (top), and following 5-days storage at 37 °C and 70%, 80% and 90% RH. (**f**) CFU change showing the effect of temperature and humidity (5-days storage) on bare membranes and NaCl, K_2_SO_4_, and KCl filters in inactivating *K. pneumoniae* after 30-min incubation (*n* = 3–10, mean ± SD). §: below detection limit. ns: *P* > 0.05; *P < 0.05; **P < 0.01; ****P < 0.0001, by *t* test.

Strain-nonspecific bacteria inactivation on NaCl, K_2_SO_4_ and KCl coated filters was tested by exposure to four further bacteria strains (MRSA*, P. aeruginosa, S. pyogenes* and *E. coli*). Similar to *K. pneumoniae*, the salt filters exhibited inactivation capabilities irrespective of pathogen strain (Fig. 3a–d). In particular, in the case of *E. coli, P. aeruginosa* and *S. pyogenes,* the bacteria showed significant decrease in CFU on all salt filters compared to bare membranes, even within 5 min (General Linear Model, *P* < 0.001 for *E. coli*, *P* < 0.001 for *P. aeruginosa*, *P* < 0.05 for *S. pyogenes*) (Fig. 3a–c). NaCl-coated filters showed effective time-dependent MRSA inactivation (General Linear Model, *P* < 0.001) (Fig. 3d). Therefore, these data indicate that, due to exploitation of a physical mechanism to inactivate the pathogens (i.e., evaporation-induced salt recrystallization), the salt-coated filters offer a universal infection prevention unit.

### Exposure of salt coatings to harsh environmental conditions

Next, the environmental stability of the salt-functionalized filters was investigated. The filters were stored at 37 °C and 70%, 80% and 90% RH for 5 days. The SEM analysis showed that all salt types remained coated on the surface of the filter fibers (Fig. 3e). Additionally, the salt coatings showed morphological changes and increased roughness due to recrystallization at the conditions above the respective critical RH (i.e., at 80% and 90% RH for NaCl, and at 90% RH for KCl). The salt-functionalized filters retained their ability to inactivate *K. pneumoniae* (Fig. 3f). Interestingly, the salt-coated filters showed a significant increase in inactivation properties after exposure to the humid environment, and no bacteria were detected from the filters after storage at 90% RH (*t* test, *P* values on graph). This phenomenon is due to the morphological change of the salt coatings after storage, leading to increased surface roughness which affects viability of bacteria^21^. Additionally, bare membranes showed the opposite trend, where the bacteria recovered from filters stored at higher RH levels yielded significantly higher CFU counts as compared to the ambient condition (*t* test, *P* values on graph). This is due to remaining humidity trapped in the membranes, protecting the bacteria. Altogether, these findings indicate that prolonged exposure of the salt-coated filters to harsh environmental conditions does not compromise the stability of the coating, while inducing further antimicrobial activity. Notably, two important conclusions come from these findings: (i) the salt-based coatings can be safely used at different environmental conditions of use and storage, and (ii) the salt-coated filters will remain highly effective against pathogens after exposure to the humidity levels generated while breathing, ensuring reusability of the filters.

## Conclusion

In summary, we developed an antimicrobial respiratory protective system with high filtering performance and improved breathability levels compared to regular masks, based on the salt functionalization technology of large-pore membranes. The filters coated with different salt types are able to capture more pathogens than the bare membranes, converting a non-functional system into an active respiratory protection unit. Since the infectious aerosols tested in this work have a small particle size (2.5–4 µm), high filtration efficiency can also be expected against bigger particles responsible for disease transmission through large droplets. Additionally, commercial mask filters are produced by melt-blowing; by converting the more inexpensive spunbond large-pore PP membranes into functional filters by salt coating and controlling the final mask design, the production cost of masks will be reduced. Simultaneously, the enhancement in filtration efficiency does not cause any decrease in the breathability of the large-pore membranes, which is not achievable by traditional technologies. Due to the natural salt recrystallization process physically neutralizing both Gram-positive and Gram-negative bacteria within a short time, the salt-coated filters offer broad-spectrum protection. This results in no risk of cross infection, safe reusability of the device without further processing, reduced amounts of biohazardous waste and no risk of shortage of respirators during outbreaks. Additionally, the salt coatings remained stable on the filters following prolonged exposure to high temperature and humidity. This offers complete protection at varying environmental conditions of use or storage as well as ensures the recyclability of the filters. Overall, the results indicate that our technology can be used to fabricate respiratory protective devices with high filtration and breathability performance, which achieve strain-nonspecific protective efficacy. Notably, the simple and cost-effective functionalization process with salt addresses all major technical challenges of respiratory protection devices in a single system and could be readily applied to other devices that are already in use, such as filters in buildings and hospitals. This comprehensive technology could lead to an enhanced and timely response to epidemics and pandemics, such as the case of COVID-19, and provides a safe and effective solution to prevent diseases globally.

## Methods

### Filters preparation

Large-pore membranes were obtained from three-ply surgical masks (Fisherbrand Facemasks; Fisher Scientific, Pittsburgh, PA, USA). The middle membrane (active filtration unit) and outer protective membrane were discarded; the innermost PP membrane (typically used against the wearer’s face for mechanical protection of the middle filter; ~ 25 g/m^2^) was used to produce the salt filter samples. As the innermost mask membrane has higher porosity than the middle filter, it was selected for the study; by stacking different numbers of layers, the performance of the salt filter samples was controlled by varying the thickness. Circular samples (3-cm radius) of the mask innermost large-pore membrane were cut (bare membranes, labelled Bare × # where # is the number of stacked layers). The membranes were coated with different salt types to obtain the salt-functionalized filters: sodium chloride (NaCl; Sigma Aldrich, St. Louis, MO, USA), potassium sulfate (K_2_SO_4_; Sigma Aldrich), and potassium chloride (KCl; Sigma Aldrich). To prepare the coating solutions, the salts were dissolved in filtered (0.22 µm pore size; Corning, Tewksbury, MA, USA) DI water under stirring at 400 rpm and 90 °C for NaCl, and 400 rpm and room temperature (RT) for K_2_SO_4_ and KCl (final salt concentrations: 29.03 w/v%, 9.72 w/v% and 26.31 w/v% for NaCl, K_2_SO_4_ and KCl, respectively). Surfactant (Tween 20, Fisher Scientific) was added at 1 v/v%. The salt filters were prepared by completely pre-wetting the membrane samples with ~ 350 μL of a given coating solution (pre-wet membranes). The amount of coated salt was controlled by varying the volume of coating solution (0, 300, 600, or 1,200 µL) in which the pre-wet membranes were deposited in 60 × 15 mm petri dishes; more layers of pre-wet membrane samples were added on top based on the desired total number of layers (1, 3 or 5), and any air bubbles were carefully removed. The salt filter samples were dried overnight in an incubator (Thermolyne 42000; Thermolyne, Dubuque, IA, USA) at 45 °C. The salt-coated filter samples were labelled Salt × #_vol_, where Salt is the salt type (NaCl, K_2_SO_4_, or KCl), # the number of stacked layers (1, 3 or 5), and _vol_ the volume of coating solution in which the filters were dried (0, 300, 600, or 1,200 µL).

### Bacteria cultures

*Klebsiella pneumoniae* (ATCC BAA-1705), methicillin-resistant *Staphylococcus aureus* (ATCC 33593), *Escherichia coli* (ATCC 25922), *Pseudomonas aeruginosa* (ATCC 10145), and *Streptococcus pyogenes* (ATCC 19615) were streaked and grown in appropriate agars and growth media, respectively. The bacteria cultures were grown following standard practice. The detailed procedures are in the Supplementary Methods. The cultures were washed 3 times in phosphate buffered saline (PBS) before experiments.

### Filtration efficiency tests

Filtration efficiency tests were designed by adapting the ASTM F2101-14/19 standard tests^22,23^ recommended by the Food and Drug Administration (FDA)^24^. The detailed operation of the filtration efficiency test apparatus is in the Supplementary Methods. Briefly, the filter samples (4.9 cm^2^ exposed area) were exposed to 60 µL of aerosolized (diameter = 2.5–4 μm) *K. pneumoniae* DI water suspension (OD_600_ = 10), under an air flow rate of 0 or 15 Lpm.

The bacteria were reconstituted from the filter samples as follows. The filter samples were incubated in PBS for 30 s–1 min. After vortexing, the filters were centrifuged (6,000 rpm, RT, 1 min) in a new tube to collect any remaining bacteria. The recovered bacteria were centrifuged (14,000 rpm, 15 min, 4 °C) to discard any salt/surfactant dissolved from the filters, and then resuspended in 1.2 mL of fresh PBS, eliminating any interference with assays.

The total amount of bacteria contained in the exposure aerosols was determined by aerosolizing 60 µL of *K. pneumoniae* suspension into a 15-mL tube containing 1.5 mL of PBS for 30 s, followed by 1-min aerosolization of DI water to avoid drying of the bacteria condensed against the tube wall. After vortexing, the bacteria were centrifuged (14,000 rpm, 15 min, 4 °C) and resuspended in 1.2 mL of fresh PBS.

The filtration efficiency was calculated as the ratio of the amount of bacteria recovered from the filter sample to the total amount of bacteria contained in the exposure aerosols. The amount of bacteria was determined as the protein concentration measured with bicinchoninic acid assay (Micro BCA protein assay kit; Thermo Fischer Scientific, Waltham, IL, USA), with bovine serum albumin (BSA) standard.

### Pressure drop tests

Pressure drop tests were designed by adapting the MIL-M-36945C standard test^25^, recommended by the FDA^24^. The detailed operation of the pressure drop test apparatus is in the Supplementary Methods. Differential pressure measurements were conducted at an air flow rate of 8 Lpm (breathing condition^26^) for 15 s, with (P_1_) and without (P_0_) loaded filter samples. The final pressure drop was calculated as ΔP = (P_1_ – P_0_)/A, where A is the area of filter sample exposed to the air flow (6.6 cm^2^). The quality factors (QF) of the bare membranes and salt-coated filters were calculated at 15 Lpm (QF = – ln(1 – F))/(P_1_ – P_0_), where F is the fraction of captured bacteria^1^); notably, filtration efficiency and pressure drop tests were conducted separately.

### Test of bacteria stability change on filters in vitro

Aerosols of multiple bacteria were exposed to the bare membranes (3 layers) and salt-functionalized filters (3 layers; NaCl, K_2_SO_4_, KCl). After washing, the bacteria were resuspended in DI water to an OD_600_ of 12.5 (*K. pneumoniae, P. aeruginosa* and *E. coli*) or 100 (MRSA and *S. pyogenes*). Highly concentrated bacteria stocks were used to ensure that the bacteria recovered from the filters were detectable, due to the low filtration efficiency of the bare membranes and the inactivation of the bacteria occurring on the salt filters. For reference, the *K. pneumoniae* stock used is 5 × 10^8^ CFU/mL, as opposed to the 3 × 10^0^–5 × 10^1^ CFU/mL of bacteria detected in hospitals (assuming 50% RH indoors)^27^.

The nebulizer unit was placed on top of the filter samples (radius = 1.2 cm), which were loaded on a porous support, and 20 μL of bacteria suspension were aerosolized (30 s). After 3, 5, 15 or 30 min incubation on the filter samples, the bacteria were reconstituted as described above. After removing the supernatant containing salt/surfactant, the bacteria were resuspended in 100 μL of PBS. The amount of bacteria contained in the exposure aerosols (0 min incubation on the filters) was determined by aerosolizing 40 µL of bacteria suspension into a 15-mL tube, similar to above; the bacteria were resuspended in 100 μL of PBS. CFU measurements were obtained by incubating 5 μL of 10 × dilutions onto MH II agar plates (*K. pneumoniae,* MRSA and *E. coli*), TSA plates (*P. aeruginosa*) or BHI agar plates (*S. pyogenes*) at 37 °C overnight. CFU were divided by the amount of bacteria recovered from the filters (determined as protein concentration). The measurements were expressed relative to the CFU at 0 min incubation on the filters (Relative CFU = CFU_sample_/CFU_0 min_).

### Test of bacteria stability change on filters in vivo

20 μL of *K. pneumoniae* DI water suspension (OD_600_ = 10.5) were aerosolized on the filter samples and incubated for 5, 15 and 30 min. A lethal dose of bacteria reconstituted from the filters was used to infect 8 7-week old BALB/c mice (KOATECH, Pyeongtaek, Republic of Korea) per group by the intranasal route. As negative controls, two mice groups were infected with the bacteria before and after aerosolization, respectively. The body weight change was measured daily for 10 days. The mice were euthanized if the body weight reached below 75% of the starting weight. Kyung Hee University (KHU) Institutional Animal Care and Use Committee (IACUC), which operates under National Veterinary Research and Quarantine Service (NVRQS) and animal welfare law and regulations of the WOAH-OIE (World organization for animal health), provided approval for all animal protocols (KHUASP(SE)-18-085). The approved protocols and guidelines of KHU IACUC were followed for all animal experiments and husbandry related to this study. At day 3 post-infection, 4 mice per group were sacrificed to collect the lung tissues. The lung supernatants from the homogenizing process were used to measure OD_600_ (NanoDrop One C; Thermo Fisher Scientific).

### Performance of filters at different environmental conditions

Bare membranes (Bare × 3) and salt-functionalized filters (NaCl × 3_600_, K_2_SO_4_ × 3_600_, KCl × 3_600_) were stored in an environmental chamber (Memmert HPP260; Memmert, Buchenbach, Germany) at 37 °C, and 70%, 80% and 90% RH. The bacteria stability change on filters in vitro was tested against *K. pneumoniae* aerosols at day 5 of storage, by measuring the CFU after 30 min incubation of the bacteria on the filters, as described above.

### Electron microscopy analysis

SEM analysis (secondary electron mode at 20 kV, Hitachi S-3000N; Hitachi, Toronto, Canada) was performed on the salt filters (NaCl, K_2_SO_4_, KCl) coated with a gold layer (thickness = 10 nm), and an EDX detector (Oxford Instruments, Concord, MA, USA) was used for the EDX analysis. The pore size of bare membranes and salt filters was determined from SEM images.

TEM analysis (200 kV, JEOL JEM 2100; JEOL, Peabody, MA, USA) was performed on *K. pneumoniae* reconstituted after incubation on the bare membranes and salt-functionalized filters, as described above. The bacteria were deposited on copper grids (Electron Microscopy Sciences, Hatfield, PA, USA) and negatively stained with tungsten using a solution of 1.5 w/v% phosphotungstic acid hydrate (pH = 7.0) (Sigma Aldrich).

### Statistical analysis

Pearson correlation coefficients basis of correlations was conducted between levels of salt weight and volume. The statistical analysis was performed by using one-way analysis of variance (ANOVA), two-way ANOVA, General Linear Model, *t* test, and chi-square analysis for multiple comparisons. SPSS ver. 21 (IBM, Armonk, NY, USA) and Minitab (Minitab, State College, PA) were used. The significance of multiple comparisons was considered by *P* value; *P* value of less than 0.05 was considered significant.

## Supplementary information

Supplementary Information.

## Data Availability

All data generated or analysed during this study are included in this published article (and its Supplementary Information files).
