# Supplementary material for: Salt coatings functionalize inert membranes into high-performing filters against infectious respiratory diseases
Source: Sci Rep. 2020 Aug 17;10:13875. doi: 10.1038/s41598-020-70623-9 (PMC7431535; doi:10.1038/s41598-020-70623-9)
Supplement: Supplementary file 1 — Supplementary Information. [file 41598_2020_70623_MOESM1_ESM.pdf]

Salt coatings functionalize inert membranes into high-performing filters against infectious respiratory diseases

Supplementary Information

Ilaria Rubino[1], Euna Oh[1,2], Sumin Han[1], Sana Kaleem[1], Alex Hornig[1], Su-Hwa Lee[3], Hae-Ji Kang[3], Dong-Hun Lee[3], Ki-Back Chu[3], Surjith Kumaran[1], Sarah Armstrong[4], Romani Lalani[1], Shivanjali Choudhry[4], Chun Il Kim[4], Fu-Shi Quan[5], Byeonghwa Jeon[2,6], and Hyo-Jick Choi[1,*]

[1]Department of Chemical and Materials Engineering, University of Alberta, Edmonton, AB T6G 1H9, Canada.

[2]School of Public Health, University of Alberta, Edmonton, AB T6G 1H9, Canada.

[3]Department of Biomedical Science, Graduate School, Kyung Hee University, Seoul, 130-701, Korea.

[4]Department of Mechanical Engineering, University of Alberta, Edmonton, AB T6G 1H9, Canada.

[5]Department of Medical Zoology, Kyung Hee University School of Medicine, Seoul, 130-701, Korea.

[6]Environmental Health Sciences, School of Public Health, University of Minnesota, Saint Paul, MN 55108, USA.

[*] Correspondence to: hyojick@ualberta.ca (HJC).

Supplementary Figures

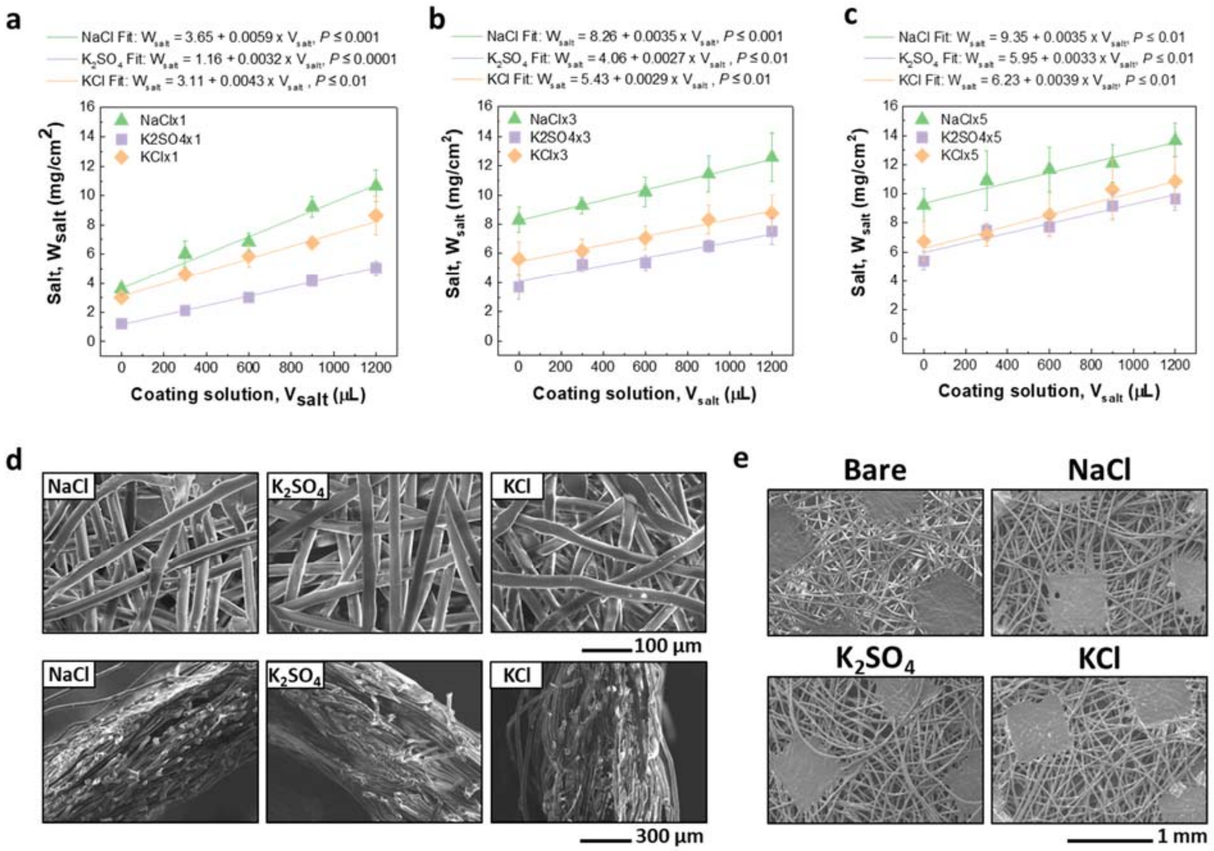

Supplementary Figure S1. Production of salt-coated filters. (a-c) Relationship between volume of salt coating solution in which the filters were dried (V_{salt}) and amount of salt coated on the filters (W_{salt}) for 1 **(a)**, 3 **(b)**, and 5 **(c)** stacked layers (*n* = 5, mean ± SD). Linear fit equations are shown. **(d)** SEM images of NaCl, K_2SO_4, and KCl filters. Top: plain view, bottom: cross-sectional view. **(e)** SEM images of Bare×1, NaCl×1_{600}, K2SO4×1_{600}, and KCl×1_{600}.

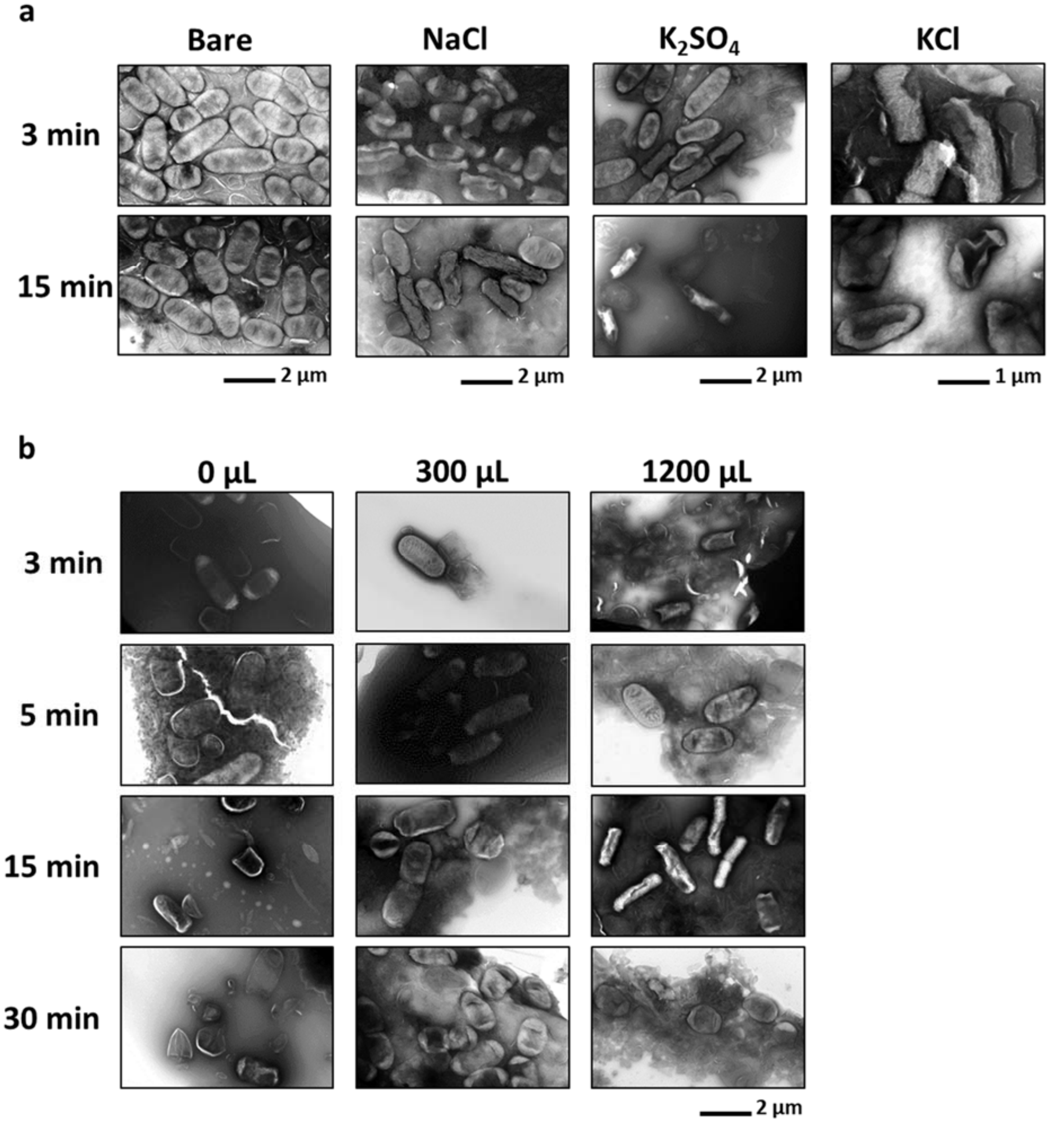

Supplementary Figure S2. TEM images of *K. pneumoniae* after incubation on salt-coated filters. (a) TEM images of *K. pneumoniae* incubated on Bare×3, NaCl×3_{600}, K2SO4×3_{600}, and KCl×3_{600} for 3 and 15 min. **(b)** TEM images of *K. pneumoniae* incubated on NaCl filters coated with different amount of salt for 3, 5, 15 and 30 min.

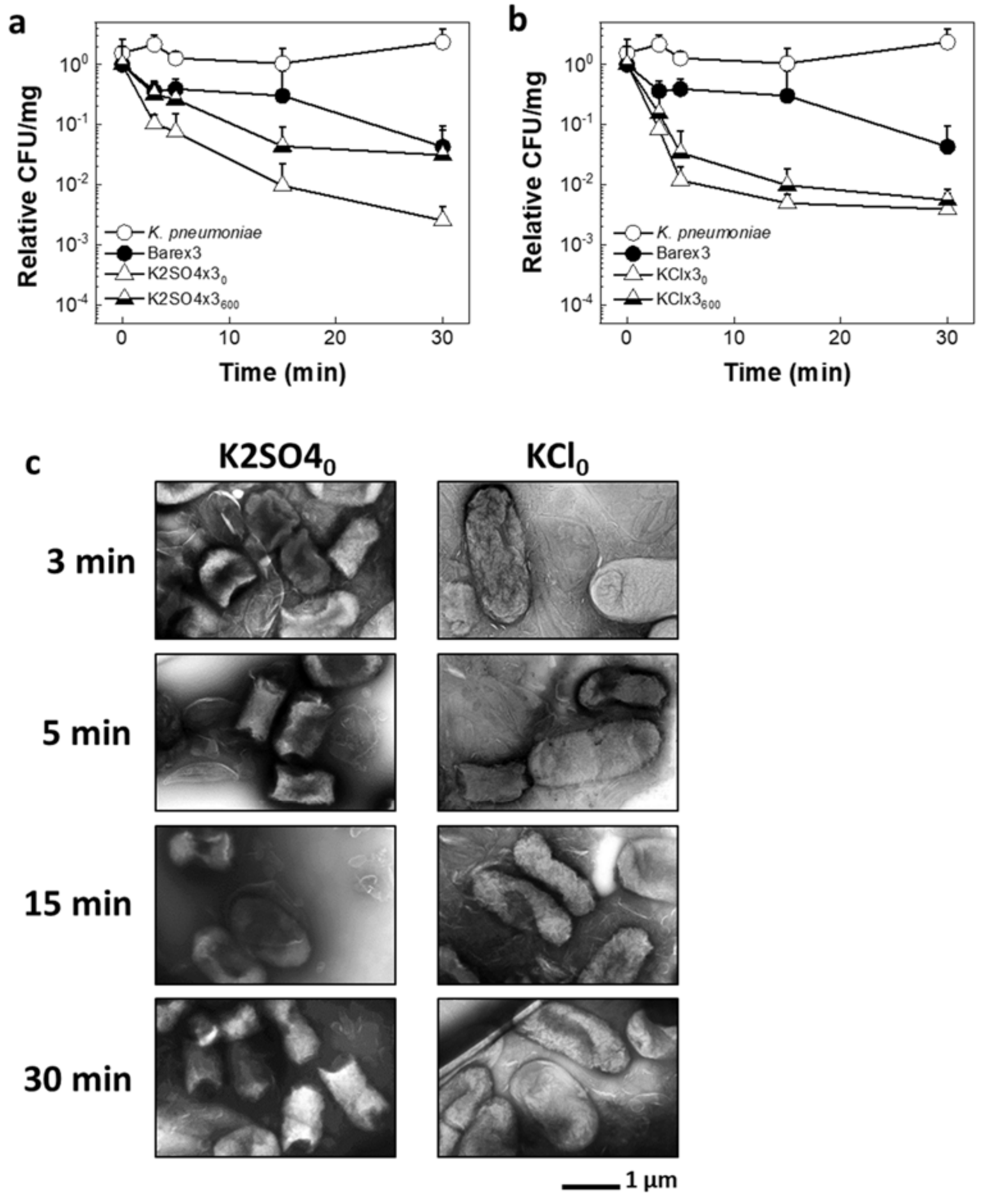

Supplementary Figure S3. Effect of *K. pneumoniae* incubation on K_2SO_4 and KCl filters coated with different amount of salt. (a,b) CFU change showing the effect of incubation time on *K. pneumoniae* exposed to K_2SO_4 **(a)** and KCl **(b)** filters coated with different amount of salt (n = 5–38, mean ± SD). **(c)** TEM images of *K. pneumoniae* incubated on K2SO4×3_0 (left) and KCl×3_0 (right) filters for 3, 5, 15 and 30 min.

Supplementary Tables

Supplementary Table S1. Quality factors values for bare membranes and salt-coated filters.

Condition	Quality factor (Pa^{-1})
Bare×1	0.026
$NaCl_{600}$×1	0.054
$K2SO4_{600}$×1	0.033
KCl_{600}×1	0.053
Bare×3	0.012
$NaCl_{600}$×3	0.014
$K2SO4_{600}$×3	0.015
KCl_{600}×3	0.019
Bare×5	0.008
$NaCl_{600}$×5	0.010
$K2SO4_{600}$×5	0.013
KCl_{600}×5	0.012

Supplementary Table S2. Pore sizes of bare membranes and NaCl, K_2SO_4 and KCl filters (1 layer, 600 μL).

Condition	Pore size (μm)
Bare	62 ± 38
NaCl	49 ± 29
K_2SO_4	52 ± 31
KCl	49 ± 29

Supplementary Methods

Bacteria cultures procedures. *Klebsiella pneumoniae* (ATCC BAA-1705) was streaked on tryptone soya agar (TSA; Oxoid, Nepean, Ontario, Canada) plates and incubated at 37 °C for 24 h. Single colonies were inoculated in tryptic soy broth (TSB; BD, Franklin Lakes, NJ, USA) at 37 °C and 200 rpm for 24 h. The *K. pneumoniae* suspension was transferred into fresh TSB 1:500 and incubated at 37 °C and 200 rpm until it entered the exponential growth phase. The *K. pneumoniae* suspension was stored at -80 °C in 20% glycerol (Fisher Scientific). For experiments, *K. pneumoniae* from frozen state was grown at 37 °C and 200 rpm with Mueller Hinton (MH) broth (BD) at 1:500 to OD_{600} of 0.8.

Methicillin-resistant *Staphylococcus aureus* (ATCC 33593) and *Escherichia coli* (ATCC 25922) were streaked on MH II agar (BD) plates, *Pseudomonas aeruginosa* (ATCC 10145) was streaked on TSA plates, and *Streptococcus pyogenes* (ATCC 19615) was streaked on brain heart infusion (BHI; BD) agar plates. The plates were incubated at 37 °C overnight. Single colonies were inoculated in MH broth, TSB or BHI medium at 37 °C and 200 rpm overnight. The bacteria suspensions were transferred into fresh media 1:100 and grown at 37 °C and 200 rpm for 4 h. All bacteria cultures were washed 3 times in phosphate buffered saline (PBS) before experiments.

Operation of the filtration efficiency test apparatus. The filtration efficiency test apparatus includes a filter holder which has a top circular aperture to tightly accommodate the nebulizer unit (Aeroneb Lab Nebulizer System; Aerogen, Galway, Ireland), an air inlet open to the atmosphere above the filter samples, and an outlet below the filter samples. The filter samples are clamped in place below the nebulizer, with an area of 4.9 cm^2 exposed to the air flow. For bare membranes, the multiple layers were glued together (at the edge area not exposed to the air

flow), to facilitate handling during loading/unloading into the filter holder; salt filters (NaCl, K_2SO_4, KCl) had a thin layer of silicone (Dowsil 732 Multi-Purpose Sealant Silicone; Dow, Midland, MI, USA) at the edge area not exposed to the air flow, to secure sealing when the filters are clamped in place. A vacuum pump (WOB-L Pump 2546, capacity 45 L/min; Welch, Niles, IL, USA) was used to generate the air flow and a flowmeter with a needle valve (Omega FLD1201; Omega, Norwalk, CT, USA) was used to control the air flow rate; a bleach trap and a safety filter were placed between the filter holder and the pump. After loading the filter samples, 60 µL of *K. pneumoniae* DI water suspension (OD_{600} = 10) were added into the nebulizer unit, and the filter samples were exposed to the bacteria aerosols (diameter = 2.5–4 µm) for 30 sec, under an air flow rate of 15 Lpm. The air flow was maintained for an additional 1 min following aerosol generation, and then the filter samples were unloaded. Notably, in the case of tests run at air flow rate of 0 Lpm, no air flow was generated, and 20 µL of *K. pneumoniae* suspension were aerosolized for 15 sec at 20 sec intervals for three times (total aerosolized volume = 60 µL) to avoid aerosol condensation. Before soaking the filter samples in PBS to reconstitute the bacteria, the glue (bare membranes) or the silicone (salt-coated filters) at the sample edge were cut and discarded.

Operation of the pressure drop test apparatus. The pressure drop test apparatus includes a filter holder which has (i) an inlet above the filter samples, connected to the flowmeter (model 150 mm Correlated Flowmeter; Cole-Parmer, Vernon Hills, IL, USA), which in turn has an air inlet open to the atmosphere, and (ii) an outlet below the filter samples, connected to the needle valve used to control the air flow rate and the vacuum pump (WOB-L Pump 2522, capacity 22 L/min, Welch) used to generate the air flow. A differential pressure gauge (Traceable Manometer Pressure/Vacuum Gauge model CON3460, Fisher Scientific) is connected in parallel

to the filter holder to measure the pressure drop across it. Salt-coated filters (NaCl, K_2SO_4, KCl) had a thin layer of silicone at the edge area not exposed to the air flow, to secure sealing when the filters are clamped in place.
